# Heat shock protein 70 enhances viral replication by stabilizing Senecavirus A nonstructural proteins L and 3D

**DOI:** 10.1186/s13567-024-01414-7

**Published:** 2024-12-18

**Authors:** Lei Hou, Penghui Zeng, Zhi Wu, Xiaoyu Yang, Jinshuo Guo, Yongyan Shi, Jiangwei Song, Jianwei Zhou, Jue Liu

**Affiliations:** 1 College of Veterinary Medicine, Yangzhou University, Yangzhou, China; 2 Jiangsu Co-Innovation Center for Prevention and Control of Important Animal Infectious Diseases and Zoonoses, Yangzhou University, Yangzhou, China; 3 Beijing Key Laboratory for Prevention and Control of Infectious Diseases in Livestock and Poultry, Institute of Animal Husbandry and Veterinary Medicine, Beijing Academy of Agriculture and Forestry Sciences, Beijing, China

**Keywords:** Hsp70, SVA L and 3D proteins, stability, Substrate binding domain (SBD), SVA replication

## Abstract

Senecavirus A (SVA) is an emerging pathogen that causes idiopathic vesicular infections in pig herds, posing a potential threat to their production performance. Heat shock protein 70 (Hsp70) is a molecular chaperone that plays an important role in host homeostasis under both physiological and stress conditions. However, the effects of Hsp70 on SVA infection and its underlying regulatory mechanisms remain unclear. Here, we confirmed that Hsp70 expression promotes SVA infection, as evidenced by the expression of viral proteins, viral titers, and the number of rSVA-eGFP-infected cells. This positive regulatory role of Hsp70 is mainly involved in post-entry stages of SVA. Viral proteins that interacted with Hsp70 were screened, and co-immunoprecipitation (co-IP) shows an interaction between Hsp70 and SVA L and 3D proteins. Subsequently, we determined that the expression of Hsp70 is beneficial for the stability of the SVA L and 3D proteins. Additionally, the substrate-binding domain (SBD) of Hsp70 plays an important role in the interaction between Hsp70 and SVA L or 3D proteins; and the deletion of this domain results in the loss of the stabilizing effect of Hsp70 on SVA L and 3D proteins and the positive regulatory effect of Hsp70 on SVA replication. These results reveal that Hsp70 promotes SVA infection by stabilizing viral L and 3D proteins and provides a strategy for preventing and controlling SVA infection.

## Introduction

Senecavirus A (SVA), formerly known as Seneca Valley virus (SVV), is a single-stranded, positive-sense, non-enveloped RNA virus that belongs to a single representative species of the genus *Senecavirus* in the *Picornaviridae* family [1]. SVA was first isolated in a cell culture contaminant in 2002, and its complete genome sequence is closely related to that of *Cardiovirus* [1]. The SVA genome encodes a single polyprotein that is processed into structural proteins (VP4, VP2, VP3, and VP1) that compose the viral capsid and nonstructural proteins (2A, 2B, 2C, 3A, 3B, 3C, and 3D) involved in viral replication, by viral proteases [1]. SVA infection in swine has been reported in many countries and is mainly characterized by fluid-ruptured vesicles and ulcerative lesions on the snout or hooves [2–4]. Since the first identification of Chinese strains of SVA in 2015, an increasing number of SVA infections have been reported in many provinces in China, indicating that SVA has spread widely [5, 6]. However, to date, no specific or effective treatment for SVA has been clinically applied. Understanding the molecular mechanisms underlying viral replication is crucial for preventing and controlling SVA infections.

Viral replication is closely related to the cellular factors of the host [7]. Viruses hijack and manipulate host proteins to support their entire life cycle. Heat shock proteins (Hsps), also known as molecular chaperones, are highly conserved proteins that play an important role in cellular homeostasis under physiological and stress conditions [8]. They mainly focus on maintaining protein stability and functionality and are involved in immune responses and inflammation [9]. Because of the absence of Hsps in viral components, viruses rely on cellular Hsps for viral protein folding and stability, unlike bacteria and eukaryotes [9]. This may be a survival strategy of the virus to eliminate cellular stress [10, 11]. Hsps are divided into many families according to their molecular weights such as Hsp10, Hsp40, Hsp60, Hsp70, and Hsp90 [12], which determine differences in their locations and functions [13].

Hsp70, a 70-kDa molecular chaperone, is necessary for the folding and refolding of newly synthesized or misfolded proteins [14, 15] and the assembly of protein complexes [16]. Hsp70 exhibits different regulatory relationships during viral infections. Zika virus (ZIV), enterovirus 71 (EV71), cucumber necrosis virus (CNV), and porcine reproductive and respiratory syndrome virus (PRRSV) interact with or recruit Hsp70 through their viral proteins which improved viral infection [17–20]. In contrast, Hsp70 exhibits antiviral responses against certain viruses. For example, the ability of influenza A virus (IAV) polymerase to bind to viral RNA was blocked by Hsp70 [21] and Hsp70 induced type I interferon production, thereby inhibiting measles virus (MeV) replication [22]. Hsp70 participates in multiple stages of the viral life cycle, including attachment [23], entry [22], uncoating [24], replication [25], and assemblage [26], however, the effect of Hsp70 on SVA infection and its underlying regulatory mechanisms remain unknown.

In this study, we evaluated the effect of Hsp70 on SVA infection, revealing that the expression of Hsp70 positively regulated SVA infection, and that this regulatory role mainly focuses on the SVA replication stage. We further screened for viral protein(s) responsible for interacting with Hsp70 and confirmed the interaction between SVA L or 3D and Hsp70, with the substrate-binding domain (SBD) of Hsp70 being crucial for this interaction and beneficial for viral protein stability. Additionally, we determined that the SBD of Hsp70 plays a positive regulatory role in SVA replication. These results clarify the mechanism by which Hsp70 is conducive to viral infection, and identify a potential target for regulating SVA replication.

## Materials and methods

### Cells, viruses, and antibodies

PK-15 and HEK-293 T cells were originally obtained from the American Type Culture Collection (ATCC) and cultured in Dulbecco modified Eagle medium (DMEM; Gibco, NY, USA) containing 5–10% fetal bovine serum (FBS) (Gibco, Life Technologies, USA), streptomycin, and penicillin at 37 °C in a 5% CO_2_ incubator. SVA CHhb17 strain and an anti-SVA VP1 monoclonal antibody, which was preserved in our laboratory, was used in this study. Other antibodies were purchased commercially and included: rabbit anti-GFP antibody (ET1602, HuaAn), mouse anti-β-actin antibody (D191047, Sangon), rabbit anti-Hsp70 antibody (A20819, ABclonal), mouse anti-Flag antibody (F4049, Sigama), mouse anti-Myc antibody (05-419, Merk), horseradish peroxidase (HRP)-conjugated anti-rabbit and -mouse secondary antibodies (A0545 or A9044, Sigma), tetramethylrhod amine isothiocyanate (TRITC)-conjugated rabbit anti-mouse (ab6799, Abcam).

### Chemical reagents

VER-155008 (HY-10941, MedChemExpress), cycloheximide (CHX) (HY-12320, MedChemExpress) and 4',6-diamidino-2-phenylindole (DAPI) (D1306, Invitrogen) were obtained from Sigma-Aldrich. Chemical reagents were dissolved in dimethyl sulfoxide (DMSO) or ultrapure water in accordance with the manufacturer’s recommendations.

### Viral infection and 50% tissue culture infectious dose (TCID_50_) assay

PK-15 cells pretreated or untreated with chemical reagents were washed with phosphate-buffered saline (PBS) and then infected with SVA at a multiplicity of infection (MOI) of 1 for 1 h at 37 °C. After removing the unbound virus, whole-cell culture medium was collected at the indicated times from SVA-infected cells and assayed for viral titers by serial dilution. The monolayer PK-15 cells seeded into 96-well cell culture plates were inoculated with 100 μL of tenfold serial dilutions of samples and tested in eight replicates. The cells were cultured until cytopathic effects were observed. TCID_50_ values were determined using the Spearman and Karber method [27].

### Cell viability assay

PK-15 cells were pretreated with various concentrations of VER-155008 and cell viability was measured using the MTT Cell Proliferation and Cytotoxicity assay kit (C0009M; Beyotime) at the indicated time points according to the manufacturer’s protocols.

### Plasmids construction

All plasmids encoding various viral proteins labelled with GFP used in this study were stored in our laboratory. The cDNA of *Hsp70* were amplified using reverse-transcription polymerase chain reaction (RT-PCR) and then subcloned into pCMV-Flag plasmids. All generated mutant plasmids of *Hsp70* and viral *3D* gene were corrected by sequencing. All the primers used in plasmid construction are listed in Table 1.

**Table 1:** **Primers and siRNA in this study**

Primers and siRNA	Sequence (5’-3’)
si*Hsp70*-1	GGCCUUUCCAGGUGAUCAATT
si*Hsp70*-2	GCGCAACGUGCUCAUCUUUTT
si*Hsp70*-3	CCAAGCAGACGCAGAUCUUTT
si*Hsp70*-4	UGCACCUUGGGCUUGUCUCCGUCGU
si*Hsp70*-5	AGGCCAACAAGAUCACCAUTT
si*Hsp70*-6	GGUGCUGACCAAGAUGAAGTT
siCon	UUCUCCGAACGUGUCACGUTT
Flag-Hsp70-F	TTGTCGACTATGGCCAAAGCCGCGGCGAT
Flag-Hsp70-R	TTGGTACCCTAATCCACCTCCTCAATGGTAGGGC
Flag-Hsp70 (ΔSBD)-F	CAGGACCTGCTGCTGCTGAGCAAGGAGGAG
Flag-Hsp70 (ΔSBD)-R	CTCCTCCTTGCTCAGCAGCAGCAGGTCCTG
Flag-Hsp70 (ΔNBD)-F	ATTCGGTCGACTATGAACGTGCAGGACCTG
Flag-Hsp70 (ΔNBD)-R	AGGTCCTGCACGTTCATAGTCGACCGAATT
Myc-L-F	TTGAATTCGGATGCAGAACTCTCATTTTTCTTTCG
Myc-L-R	TTGGTACCTTACTGCAGCTCGTATACGATGTCCAG
Myc-3D-F	TTGAATTCCGATGGGACTAATGACTGAGCTAGAGCC
Myc-3D-R	TTGGTACCTTATCAGTCGAACAAGGCCCTCCATC
GFP-3D (aa1-150)-F	TTGAATTCAGGACTAATGACTGAGCTAGAGCC
GFP-3D (aa1-150)-R	TTGGTACCGTCACCGTCTAAGAATCTCTGG
GFP-3D (aa149-298)-F	TTGAATTCAGGTGACTACTCTGATCATGTCT
GFP-3D (aa149-298)-R	TTGGTACCGGCACAACCAGAGGGGAGGC
GFP-3D (aa297-463)-F	TTGAATTCATGTGCCGCGACCAGCCTGCT
GFP-3D (aa297-463)-R	TTGGTACCTCAGTCGAACAAGGCCCTCCAT

### Plasmids and siRNA transfection

PK-15 cells cultured to 70–80% confluence were transfected with various plasmids using Lipofectamine 2000 (11668019, Invitrogen) according to the manufacturer’s protocols and infected with SVA, followed by analysis at the indicated time points. siRNA (Table 1) targeting the *Hsp70* gene were designed by GenePharma and transfected using Lipofectamine RNAiMAX (13778; Invitrogen) for 36 h according to the manufacturer’s protocols, followed by infection with SVA or transfection with various plasmids, and then processed and analyzed by western blotting and TCID_50_.

### RNA extraction and reverse transcription quantitative polymerase chain reaction (RT-qPCR)

Total RNA was extracted from the cells using TRIzol reagent (15596018, Invitrogen) and reverse-transcribed using HiScript III RT SuperMix (R323-01, Vazyme) to generate cDNA. The synthesized cDNA was amplified using Taq Pro Universal SYBR qPCR Master Mix (Q712-02, Vazyme) and measured at least three times using a LightCycler 96 system (Roche, Switzerland). The relative mRNA of the SVA 3D gene were calculated by a comparative cycle threshold (2^−ΔΔCT^) method and normalized to mRNA of the glyceraldehyde-3-phosphate dehydrogenase (GAPDH) gene. The primer sequences (5'–3') for SVA-3D and GAPDH in RT-qPCR were as follows: qSVA-3D-F (CCAACAAGGGTTCCGTCTTC), qSVA-3D-R (TTGGACGAATTTGCGTTTTAGA), qGAPDH-F (TCGGAGTGAACGGATTTGGC), and qGAPDH-R (TGACAAG-CTTCCCGTTCTCC).

### Co-immunoprecipitation and western blotting

HEK-293 T cells were co-transfected with pCMV-Flag, pCMV-Flag-Hsp70, or pCMV-Flag- Hsp70 mutants and pEGFP-C1, pEGFP-L, pEGFP-2B, pEGFP-2C, pEGFP-3A, pEGFP-3C, pEGFP-3D, or pEGFP-3D truncates for 24 h, followed by lysis with NP40 buffer containing phenylmethanesulfonyl fluoride (PMSF) (ST506; Beyotime), and then immunoprecipitated with anti-GFP agarose (PGA025; Lablead) or anti-Flag agarose (PFA025; Lablead) at 4 °C on a roller. The immunoprecipitates were harvested from the agarose gel and analyzed. Quantified cell lysates were subjected to sodium dodecyl sulfate–polyacrylamide gel electrophoresis (SDS-PAGE) and transferred onto nitrocellulose membranes (66485; Pall). The membranes were blocked with 5% non-fat milk, incubated with the appropriate primary and secondary antibodies, and detected using an AMERSHAM ImageQuant800 chemiluminescence imaging system (GE, USA).

### Immunofluorescence assays and confocal microscopy

PK-15 cells grown to approximately 80–90% confluence were transfected with the indicated plasmids for 12 h in 24-well culture plates in the presence or absence of rSVA-eGFP. They were then fixed with 4% paraformaldehyde, washed, and then incubated with the appropriate primary, TRITC-conjugated secondary antibodies, and DAPI. Fluorescent images were observed using an immunofluorescence microscope (IX73; Olympus) or a confocal immunofluorescence microscope ((TCS SP8 STED; Leica, Wetzlar, Germany). To quantify the colocalization signal of viral proteins and Hsp70, the confocal microscope images were imported to Image J software and analyzed by Pearson correlation coefficient.

### Protein half-life assay

Cells transfected with Myc-L or -3D were treated with CHX (100 μg/mL) for various periods of time to block protein synthesis. Proteins were extracted and analyzed by western blotting.

### Statistical analysis

Statistical differences were evaluated by one-way analysis of variance (ANOVA) or Student *t*-test using GraphPad Prism 9.0 software (GraphPad Software, Boston, USA). *P* value < 0.05 was considered to be statistically significant.

## Results

### SVA replication is enhanced in Hsp70-overexpressed cells

**Figure 1:**
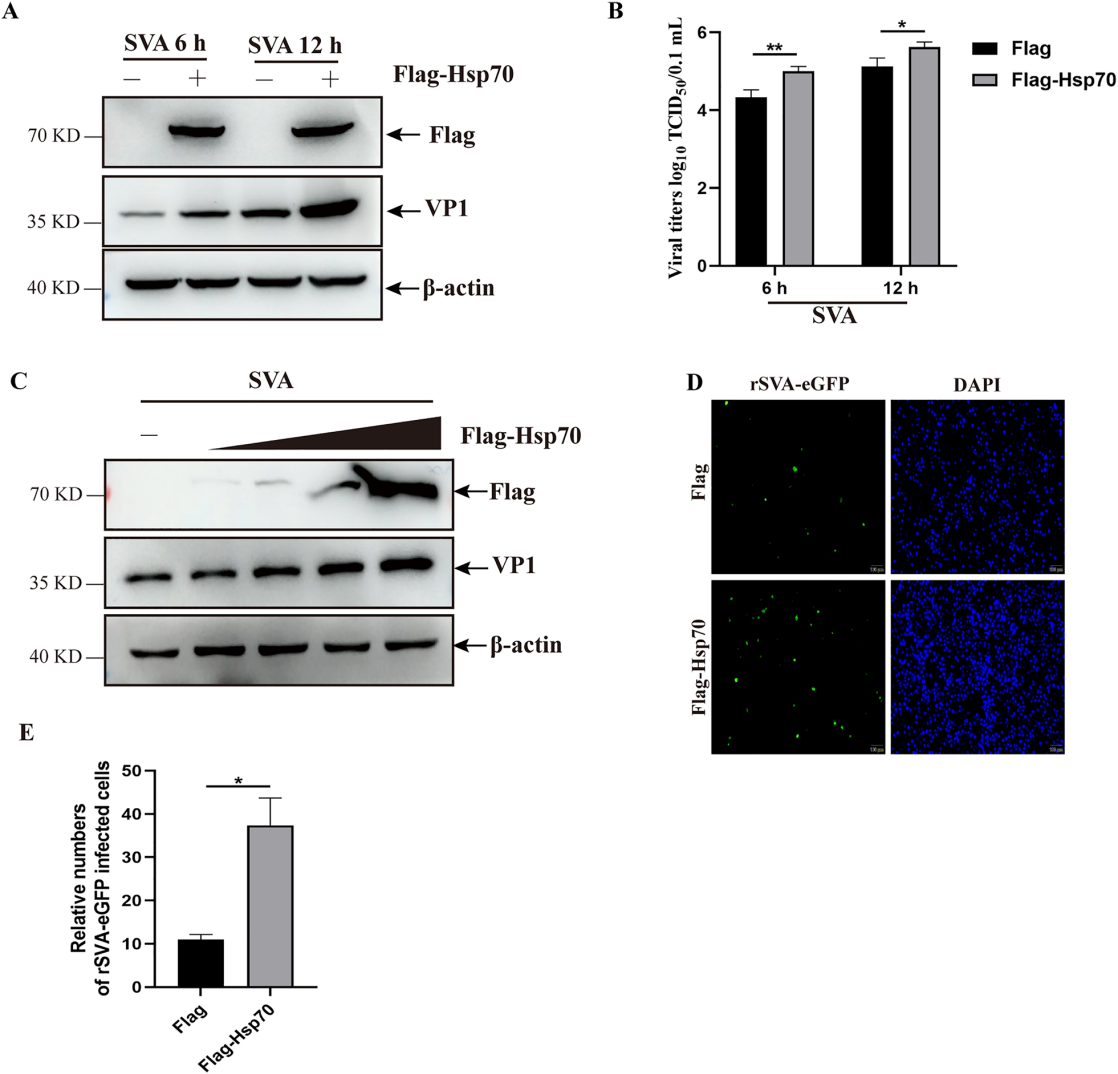
**Hsp70 promoted SVA replication.** **A** and **B** PK-15 cells transfected with Flag-Hsp70 or Flag plasmids were infected with SVA for 6 or 12 h, and the extracted proteins and whole cell culture medium were then analyzed by western blotting (**A**) and TCID_50_ analyses (**B**). **C** PK-15 cells transfected with various concentrations of Flag-Hsp70 plasmids were infected with SVA, and the extracted proteins were analyzed by western blotting. **D** and **E** PK-15 cells transfected with Flag-Hsp70 or Flag plasmids were infected with rSVA-eGFP, and the number of rSVA-eGFP-infected cells was determined by indirect fluorescence assay (IFA) (**D**). The results are presented as a histogram (**E**). The data are expressed as the means ± SD from three independent experiments (*P < 0.05; **P < 0.01).

To investigate the effect of Hsp70 on SVA replication, PK-15 cells transfected with Flag-Hsp70 plasmids were infected with SVA and analyzed using western blotting and TCID_50_. Transfection of Hsp70 into cells significantly promoted SVA replication compared to the control groups, as evidenced by an increase in viral VP1 expression and viral titers (Figures 1A and B). Measurements of SVA proliferation in cells transfected with various concentrations of Flag-Hsp70 plasmids showed a substantial increase in VP1 expression, accompanied by an enhancement of Hsp70 expression (Figure 1C). Additionally, PK-15 cells were infected with rSVA-eGFP to evaluate the effects of Hsp70 on viral replication. The number of rSVA-eGFP-infected cells, indicated by green fluorescence signals, significantly increased in Hsp70-overexpressed cells (Figures 1D and E). These data reveal that Hsp70 overexpression promotes SVA replication.

### Hsp70 silencing inhibited SVA replication

**Figure 2:**
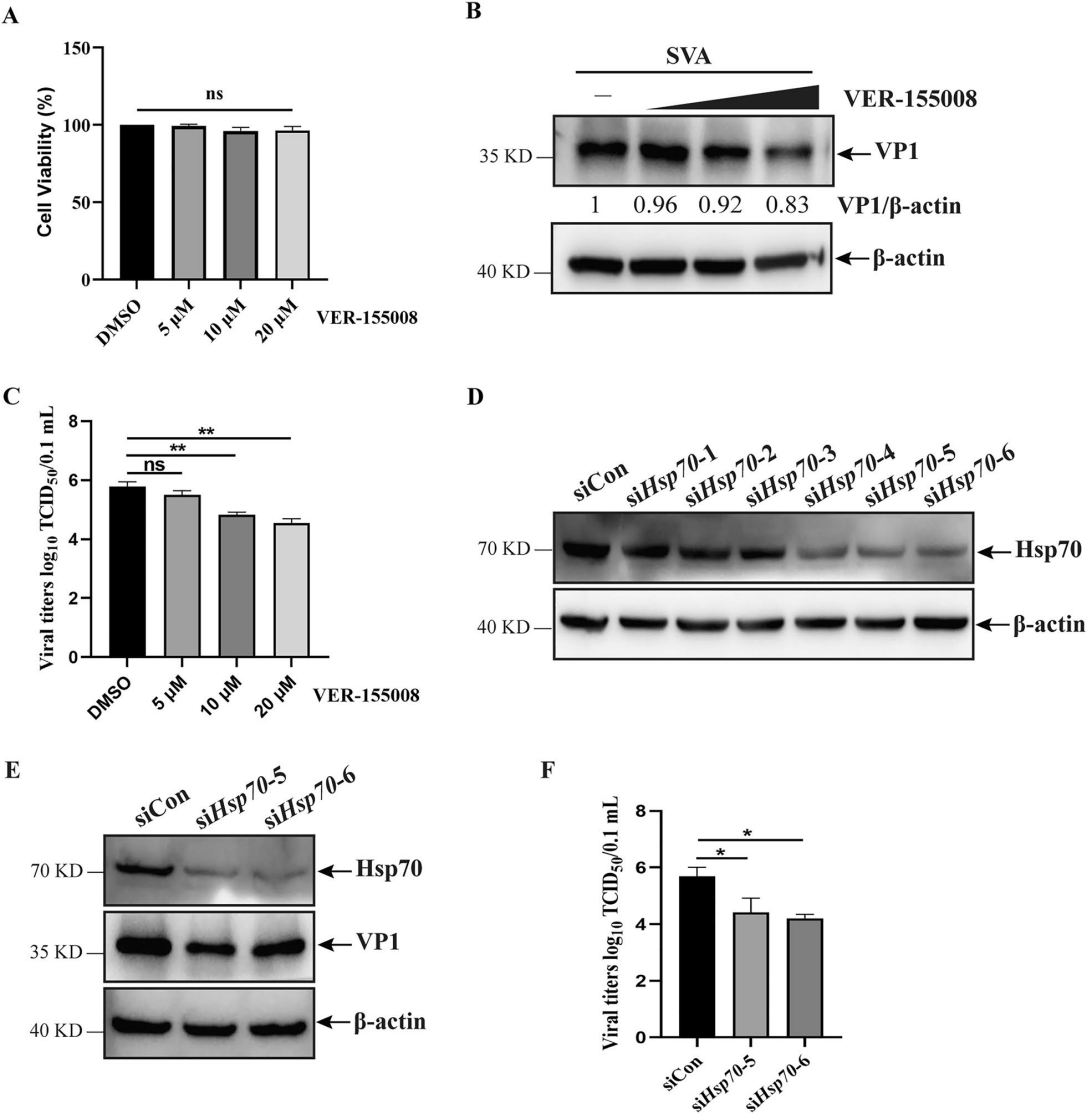
**Hsp70 silencing inhibited SVA replication. A** The detection of cell viability in PK-15 cells treated with various concentrations of VER-155008. **B** and **C** PK-15 cells treated with various concentrations of VER-155008 were infected with SVA for 12 h, and the extracted proteins and whole cell culture medium were then analyzed by western blotting (**B**) and TCID_50_ analyses (**C**). **D** The silencing effects of various siRNA on Hsp70 expression was detected by western blotting. **E** and **F** PK-15 cells treated with si*Hsp70*-5, si*Hsp70*-6, or siCon were infected with SVA for 12 h, and the extracted proteins and whole cell culture media were then analyzed by western blotting (**E**) and TCID_50_ analyses (**F**). The data are expressed as the means ± SD from three independent experiments (ns, not significant; *P < 0.05; **P < 0.01).

VER-155008, an Hsp70 inhibitor [28], was used to determine the importance of Hsp70 in SVA replication. The various concentrations of VER-155008 (5, 10, and 20 μM) did not affect cell viability (Figure 2A) and were used to analyze its regulatory effect on viral replication. Treatment with VER-155008 significantly downregulated SVA replication (Figures 2B and C). To exclude the nonspecific effects of chemical reagents on viral replication, siRNA targeting *Hsp70* were used to silence Hsp70 expression. PK-15 cells transfected with si*Hsp70*-1 to -6 show a reduction in Hsp70 expression by si*Hsp70-*4, -5, or -6 (Figure 2D). si*Hsp70*-5 and -6 were randomly selected for subsequent experiments. PK-15 cells transfected with si*Hsp70*-5 or -6 were infected with SVA and evaluated for viral replication. Viral VP1 expression and titers were significantly decreased (Figures 2E and F). These results suggest that Hsp70 positively regulates SVA replication.

### Hsp70 played an important role in the replication stage of SVA

**Figure 3:**
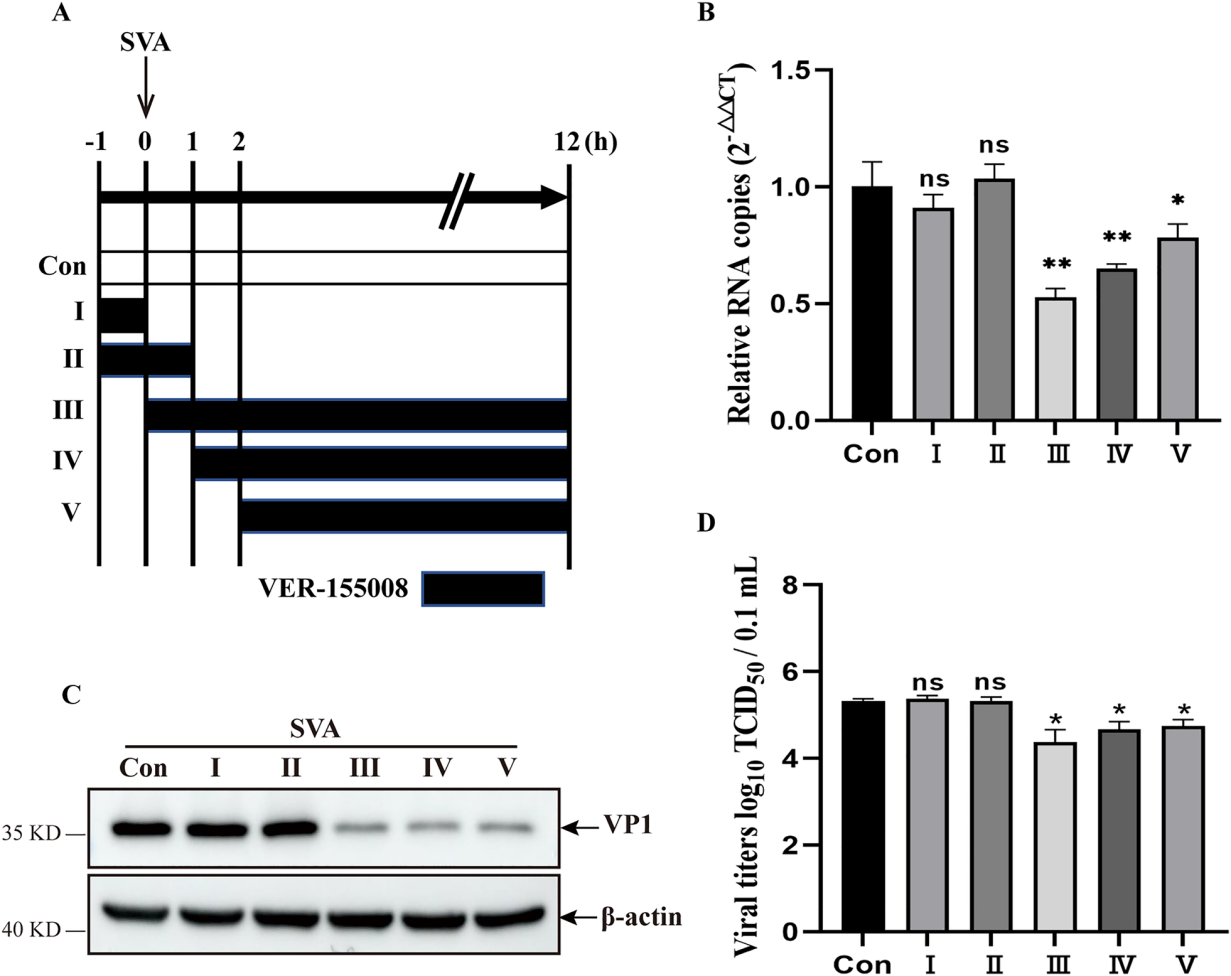
**Hsp70 was involved in the replication stage of the SVA life cycle. A** Schematic diagram of the time course of VER-155008 treatment (20 μM). The black color represents the VER-155008 treatment. **B**, **C**, and **D** PK-15 cells infected with SVA at an MOI of 1 were collected at 12 hpi and then analyzed by viral RNA copies (**B**), viral VP1 expression (**C**), and viral titers (**D**). The data are expressed as the means ± SD from three independent experiments (ns, not significant; *P < 0.05; **P < 0.01).

To determine the stage of the SVA life cycle that requires Hsp70, cells treated with VER-155008 at different time points were analyzed for SVA infection (Figure 3A). Pretreatment with VER-155008 (treatments I and II) did not affect SVA infection, as evidenced by the lack of changes in viral mRNA, VP1 expression, and viral titers, suggesting that the binding and entry stages of SVA infection were not dependent on Hsp70 (Figures 3B–D). In contrast, SVA replication was analyzed in cells treated simultaneously with VER-155008 and infected with SVA (treatment III). VER-155008 markedly suppressed viral replication and production (Figures 3B–D). After SVA infection for 1 or 2 h, the addition of VER-155008 resulted in an obvious reduction in viral mRNA, protein, and titer (Figures 3B–D). These results indicate that Hsp70 is involved in the SVA life cycle, mainly within the viral post-entry stages.

### Screening for viral proteins interacting with Hsp70

**Figure 4:**
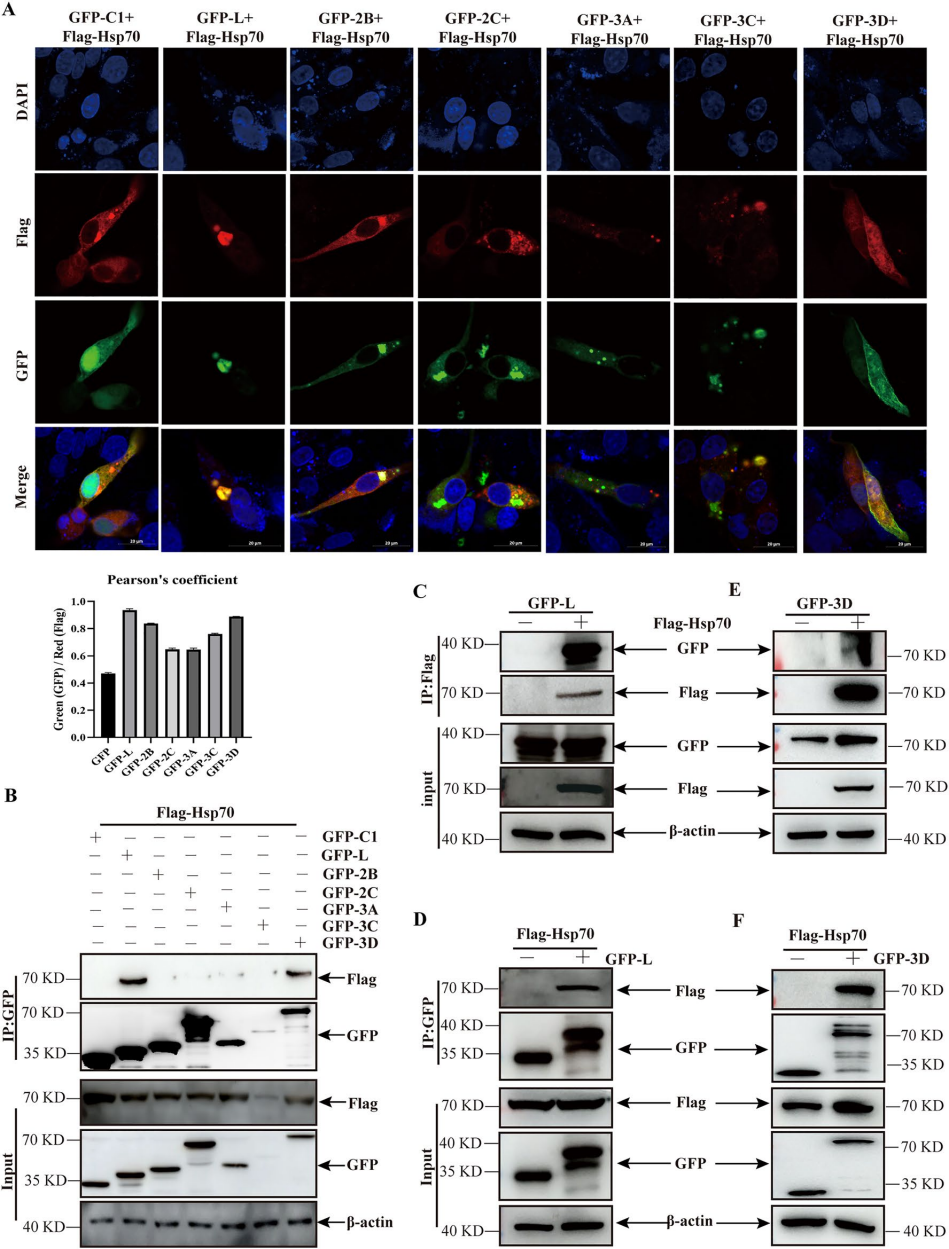
**SVA L and 3D proteins interacted with Hsp70. A** PK-15 cells were co-transfected with Flag-Hsp70 and GFP tagged various viral non-structural proteins for 24 h, followed by fixation and incubation with anti-Flag antibodies (red signals) and DAPI (blue signals); then, the cells were observed using a confocal immunofluorescence microscope. Scale bar, 20 μm. The co-localization analysis was expressed as Pearson correlation coefficient, measured for individual cells. **B** HEK-293 T cells co-expressing Flag-Hsp70 and GFP, GFP-L, GFP-2B, GFP-2C, GFP-3A, GFP-3C, or GFP-3D for 24 h were lysed and analyzed using co-IP. **C** and **E** HEK-293 T cells co-expressing Flag-Hsp70 or Flag and GFP-L (**C**) or GFP-3D (**E**) and for 24 h were lysed and immunoprecipitated using an anti-Flag antibody, followed by co-IP analysis. **D** and **F** HEK-293 T cells co-expressing Flag-Hsp70 and GFP, GFP-L (**D**), or GFP-3D (**F**) for 24 h were immunoprecipitated using an anti-GFP antibody, followed by co-IP analysis.

Viral non-structural proteins modulate viral RNA translation and replication via various strategies [29]. Based on the results of Hsp70 involvement in SVA replication, experiments were performed to determine whether SVA non-structural proteins were involved in the regulatory role of Hsp70 in SVA replication. The co-localization of various SVA nonstructural proteins and Hsp70 were analyzed by confocal microscopy in PK-15 cells, respectively. The results showed that all viral non-structural proteins completely or partially colocalized with Hsp70 (Figure 4A). Co-immunoprecipitation (Co-IP) experiments further explored the interactions between viral proteins and Hsp70. Only SVA L and 3D proteins interacted with Hsp70, respectively (Figure 4B). Subsequently, the specific interactions between SVA L or 3D and Hsp70 were validated using forward and reverse co-IP assays (Figures 4C–F). These data revealed that SVA L and 3D proteins specifically interacted with Hsp70.

### Hsp70 enhanced the stability of SVA L and 3D proteins

**Figure 5:**
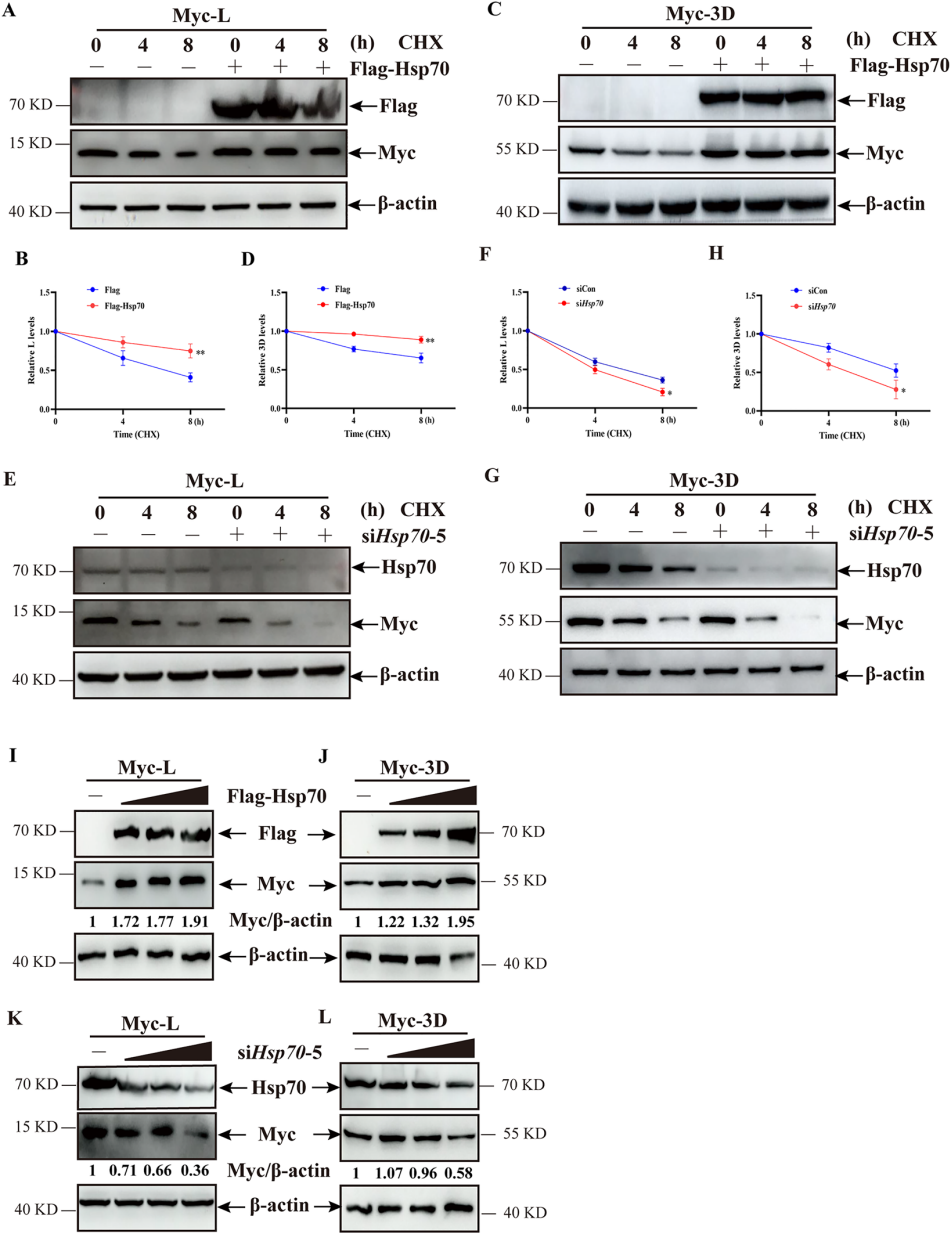
**Hsp70 reduced the half-life of SVA L or 3D proteins.** **A** and **C** PK-15 cells co-expressed with Flag or Flag-Hsp70 and Myc-L (**A**) or Myc-3D (**C**) were treated with CHX (100 μg/mL) for the indicated time durations and were then analyzed by western blotting.** B** and **D** The relative ratios of the band intensities of Myc-L (**B**) or Myc-3D (**D**) to β-actin were quantified by Image J and GraphPad software according to **A** and **C**. **E** and **G** PK-15 cells treated with si*Hsp70*-5 were transfected with Myc-L (**E**) or Myc-3D (**G**) and treated with CHX (100 μg/mL) for the indicated times, followed by analysis using western blotting.** F** and **H** The relative ratios of the band intensities of Myc-L (**F**) or Myc-3D (**H**) to those of β-actin were quantified by Image J and GraphPad software according to **E** and **G**. **I, J, K,** and **L** PK-15 cells transfected with various doses of Flag-Hsp70 or treated with various doses of si*Hsp70*-5 were transfected with Myc-L (**I** and **K**) or Myc-3D (**J** and **L**), followed by analysis using western blotting. The relative ratios of the band intensities of Myc-L or Myc-3D to those of β-actin were quantified by Image **J**. The data are expressed as the means ± SD from three independent experiments (*P < 0.05; **P < 0.01).

The positive effect of Hsp70 on SVA replication prompted us to explore whether Hsp70 mediates the stability of viral proteins. Cycloheximide (CHX), an inhibitor of protein synthesis, was used to evaluate the stability of SVA L and 3D at the indicated time points. As shown in Figures 5A and B, Hsp70 overexpression in PK-15 cells in the presence of CHX profoundly extended the half-life of SVA L compared with that in the control group. Similarly, the degradation of SVA 3D was delayed by CHX treatment in Hsp70-overexpressed cells (Figures 5C and D). Conversely, the knockdown of Hsp70 by si*Hsp70* in CHX-treated cells resulted in a significant decrease in the half-lives of SVA L and 3D (Figures 5E–H). Subsequently, the effect of different Hsp70 levels on SVA L and 3D expression was analyzed in the cells untreated with CHX. The results show that the overexpression and knockdown of Hsp70 increased and decreased SVA L and 3D expression, respectively (Figures 5I–L). These results indicate that Hsp70 mediates the stability of SVA L and 3D in cells.

### The SBD of Hsp70 is crucial for the stability of SVA L and 3D proteins and viral replication

**Figure 6:**
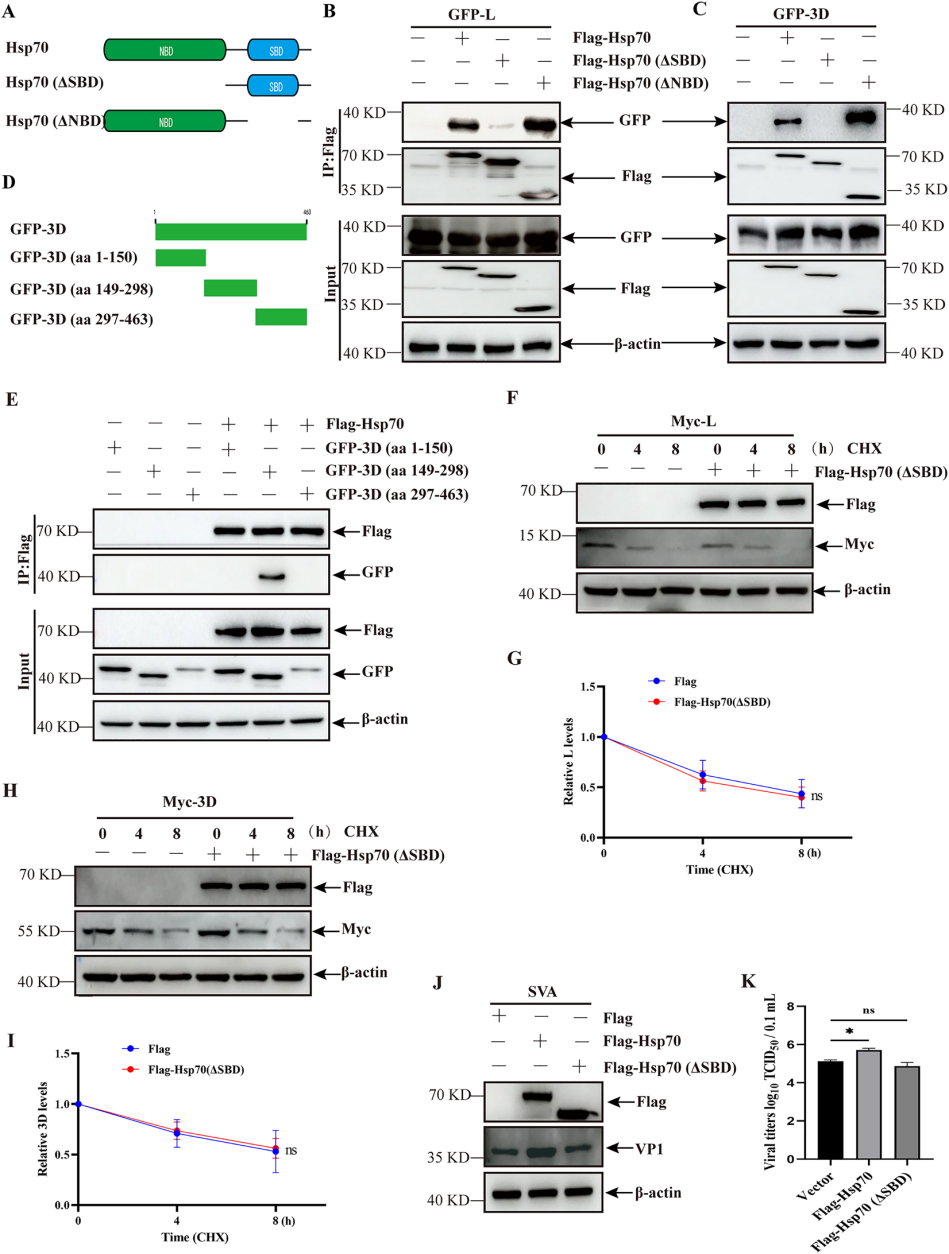
**The SBD domain mediated interactions between Hsp70 and SVA L or 3D and SVA replication.** **A** Schematic diagram of various structural domains in Hsp70. **B** and **C** HEK-293 T cells co-expressing Flag, Flag-Hsp70, Flag-Hsp70 (∆SBD), or Flag-Hsp70 (∆NBD) and GFP-L (**B**) or GFP-3D (**C**) for 24 h were lysed and immunoprecipitated using an anti-Flag antibody, followed by co-IP analysis. **D** Schematic diagram of various segments in SVA 3D. **E** HEK-293 T cells co-expressing Flag or Flag-Hsp70 and GFP-3D (aa 1–150), GFP-3D (aa 149–298), or GFP-3D (aa 297–463) for 24 h were lysed and immunoprecipitated using an anti-Flag antibody, followed by co-IP analysis. **F** and **H** PK-15 cells co-expressed with Flag- Hsp70 (∆SBD) or Flag-Hsp70 and Myc-L (**F**) or Myc-3D (**H**) were treated with CHX (100 μg/mL) for the indicated time durations, and were then analyzed using western blotting.** G** and **I** The relative ratios of the band intensities of Myc-L (**G**) or Myc-3D (**I**) to those of β-actin were quantified by Image J and GraphPad software according to F and H. **J** and **K** PK-15 cells transfected with Flag, Flag-Hsp70, or Flag-Hsp70 (∆SBD) were infected with SVA, followed by analyzing SVA replication using western blotting (**J**) and TCID_50_ (**K**) analyses. The data are expressed as the means ± SD from three independent experiments (ns, not significant; *P < 0.05).

Hsp70 contains a nucleotide-binding domain (NBD, aa 1–389) located at the N-terminal and a substrate-binding domain (SBD, aa 397–512) located at the C-terminal [30]. The plasmids expressing different domains were constructed according to the structural domains of Hsp70, such as Flag-Hsp70, Flag-Hsp70 (∆SBD), and Flag-Hsp70 (∆NBD) (Figure 6A). To determine the domain of Hsp70 responsible for the interaction with SVA L or 3D, a co-IP assay was performed in cells co-expressing GFP-L or -3D and various domains of Hsp70. As shown in Figures 6B and C, only specific bands for Hsp70 (∆SBD) and SVA L or 3D disappeared in cells, indicating that the interaction of Hsp70 and SVA L or 3D is dependent on the SBD of Hsp70. Additionally, the full-length SVA 3D protein was randomly divided into three segments (aa 1–150, aa149-298, and aa 297–463; Figure 6D), and the interaction between Hsp70 and various segments of SVA 3D was determined by co-IP. A specific band was detected in cells expressing GFP-3D (aa 149–298) (Figure 6E). These data indicate that the SBD of Hsp70 plays a crucial role in its interaction with SVA L or 3D proteins.

To explore the effect of Hsp70 SBD on the stability of SVA L and 3D, PK-15 cells co-expressed with Hsp70 (∆SBD) and SVA L or 3D were treated with CHX and analyzed by western blotting. Hsp70 (∆SBD) overexpression in PK-15 cells in the presence of CHX did not enhance the half-life of SVA L or 3D proteins compared with that in the control group (Figures 6F–I), suggesting that the stability of SVA L or 3D proteins is dependent on Hsp70 SBD. The above results prompt us to explore the effect of Hsp70 SBD on SVA replication. Hsp70 (∆SBD) overexpression in PK-15 cells did not promote SVA replication (Figures 6J and K), suggesting that with the deletion of SBD, Hsp70 lost its positive regulatory role in SVA replication compared to full-length Hsp70.

## Discussion

Viruses are specialized cell parasites that use smaller genome sizes to encode limited viral proteins and exploit the cell’s molecular machinery for genome replication, translation, and virion assembly and release [28]. Viruses have evolved strategies to manipulate cellular components or block host antiviral defenses for their own benefit. Within the cell, cellular chaperone proteins play an important role in the folding of viral proteins and protection of cells against stressors and are involved in the presentation of immune and inflammatory cytokines during viral replication [31, 32]. Furthermore, they regulate cell differentiation, survival, and death [32]. Heat shock proteins (Hsp) are a large number of chaperones that are utilized by viruses to create a favorable environment for viral survival. Hsp70 is an abundant protein in cells that directly interacts with viral polymerase to facilitate viral replication, promote the formation of a viral replication complex, and maintain the stability of viral proteins [33]. Clarifying the effect of Hsp70 on SVA infection is not only crucial for exploring antiviral targets, but also helpful for developing antiviral drugs. In this study, we explored the regulatory relationships and mechanisms between Hsp70 and SVA infection. Our results show that the expression of Hsp70 facilitates SVA replication and is dependent on the enhancement of the stability of SVA L and 3D proteins by Hsp70 through their interaction.

Hsp70 plays both positive and negative roles in various viral infections. For example, Hsp70 enhances the replication of the porcine epidemic diarrhea virus (PEDV) by interacting with membrane (M) proteins [34]. The upregulation of Hsp70 by the Japanese encephalitis virus (JEV) NS5 plays a dual role by contributing to the antagonism of type I interferon (IFN) and participating in its anti-apoptotic effects [35]. Conversely, Hsp70 inhibits influenza virus (IAV) replication by disrupting viral polymerase binding to viral RNA or by preventing the export of the ribonucleoprotein (RNP) complex to the nucleus [21, 36]. Our results reveal that overexpression and knockdown of Hsp70 significantly enhanced and reduced SVA replication, respectively, at different time points (Figures 1 and 2), suggesting that Hsp70 is an essential host factor required for SVA replication. Additionally, Hsp70 has been reported to be frequently involved in multiple stages of the viral life cycle. The binding and entry of JEV into cells are dependent on Hsp70 located on the cell surface [37]. Hsp70 is involved in the entry stage of the dengue virus (DENV) by forming a viral receptor complex [38]. In this study, compared to the control group, cells pretreated with VER-155008 and infected with SVA did not affect viral yields, whereas cells infected with SVA simultaneously or prior to VER-155008 treatment showed a reduction in SVA yields (Figure 3).

This suggests that Hsp70 is necessary for the post-entry stages of SVA rather than being involved in the binding and entry stages of SVA.

During viral infection, several host components are successfully recruited by viral proteins for viral replication, protein synthesis, and virion assembly. Hsp70 is a protein chaperone that regulates protein homeostasis under normal and stressed conditions [39]. Hsp70 regulates viral replication by interacting with viral proteins. The upregulation of Hsp70 by JEV NS5 plays a dual role in inhibiting type I IFN and apoptosis [40]. The infection of host cells with HCV can increase the expression of Hsp70, which binds to the viral replication complex and facilitates the assembly and replication of viral genes [41]. SVA non-structural proteins are essential for modulating viral RNA translation and replication [29]. Thus, we explored whether Hsp70 enhances viral replication by interacting with SVA non-structural proteins. Our results show that Hsp70 enhances the stability of SVA L and 3D proteins through the interaction between its SBD and these two viral proteins, and that deletion of SBD blocks the positive regulatory role of Hsp70 in SVA replication (Figures 4, 5, 6). Viral L protein, a papain-like cysteine protease in the *Picornaviridae* family, suppresses host antiviral properties such as shutdown of cap-dependent host protein synthesis and inhibition of host innate immune responses [42]. Viral 3D protein, an RNA-dependent RNA polymerase, facilitates the assembly of replication complexes and directly catalyzes viral RNA synthesis [43]. These two reports indicate that the Hsp70-mediated stability of SVA L and 3D proteins and enhancement of viral replication may be attributed to the long-term maintenance of the functionality of viral proteins, which needs to be studied further.

In conclusion, we demonstrate that Hsp70 is necessary for viral replication. The mechanism by which Hsp70 enhances SVA replication mainly depends on the interaction between its SBD domain and viral L and 3D proteins. Clarifying the regulatory mechanism of Hsp70 will contribute to the prevention and control of SVA infections.

## Data Availability

The datasets during and/or analyzed during the current study are available from the corresponding author on reasonable request.
